# Emergence of carbapenemase KPC-14 in an *Escherichia coli* isolate: a case report

**DOI:** 10.1093/jacamr/dlag157

**Published:** 2026-08-05

**Authors:** Leticia Fontán García-Rodrigo, María Díez Aguilar, Laura Sevillano Tripero, María Auxiliadora Semiglia Chong, Laura Cardeñoso Domingo

**Affiliations:** Microbiology and Parasitology Department, University Hospital of La Princesa, Calle de Diego de León 62, Madrid 28006, España; Microbiology and Parasitology Department, University Hospital of La Princesa, Calle de Diego de León 62, Madrid 28006, España; Microbiology and Parasitology Department, University Hospital of La Princesa, Calle de Diego de León 62, Madrid 28006, España; Microbiology and Parasitology Department, University Hospital of La Princesa, Calle de Diego de León 62, Madrid 28006, España; Microbiology and Parasitology Department, University Hospital of La Princesa, Calle de Diego de León 62, Madrid 28006, España

The introduction of new molecules, like ceftazidime/avibactam, has expanded the therapeutic options for carbapenem-resistant bacteria. Its inhibitory spectrum includes Ambler class A (like KPC), class C and certain class D β-lactamase enzymes.^1^ However, mutations in the KPC Ω-loop can confer resistance to ceftazidime/avibactam while restoring susceptibility to carbapenems.^2,3^ These variants, such as KPC-33 or KPC-14, have been described in *Klebsiella pneumoniae* following treatment with ceftazidime/avibactam, cefiderocol or imipenem/relebactam.^4^ Beyond *K. pneumoniae*, KPC-14 has also been reported in *Pseudomonas aeruginosa*.^5^

We report a case of a patient diagnosed with myelodysplastic syndrome admitted to the haematology department for a haematopoietic progenitor cell transplant. All hospitalized patients in the haematology ward undergo weekly screening for MDR microorganisms, which consists of a pharyngeal and a rectal swab. On the one hand, genotypic resistance detection is performed directly on a pool of both samples using the Vitro master diagnostica^®^ MDR Direct Flow Chip Kit (MDR kit). On the other hand, both samples are plated onto selective media: MRSA/SAID^®^ (bioMérieux) and CARB/OXA^®^ (bioMérieux) for the pharyngeal swab, and ESBL^®^ (bioMérieux), CARB/OXA^®^ and VRE^®^ (bioMérieux) for the rectal swab. One week after admission (before receiving any antibiotic therapy) the *bla*_KPC_ gene was detected in a direct sample pool of rectal and pharyngeal swabs. Subsequently, an *Escherichia coli* isolate grew on the CARB/OXA and ESBL selective plates from the rectal swab. Carbapenemase production was confirmed by immunochromatography (CORIS Biconcept O.K.N.V.I. RESIST-5), Xpert^®^ Carba-R (Cepheid^®^) and boronic acid synergy testing (ROSCOS Diagnostica). Susceptibility was determined by an automatic microdilution method (MicroScan WalkAway, Beckman Coulter), which showed resistance to carbapenems but susceptibility to ceftazidime/avibactam and cefiderocol (Table 1). Cefiderocol was tested by both the disc diffusion and broth microdilution (UMIC^®^ Bruker) methods. EUCAST breakpoints were used for interpretation (Version 15.0, 2025: https://www.eucast.org). Ten days later, the patient developed febrile neutropenia and the *E. coli* was isolated from a blood culture, leading to the initiation of treatment with ceftazidime/avibactam (12 days). The patient received ceftazidime/avibactam again at the end of the admission for 5 days due to diverticulitis and was finally discharged home. During the ceftazidime/avibactam treatment period, the KPC-producing *E. coli* was neither isolated from surveillance samples nor was the gene detected in the direct sample genotypic study. At the end of the treatment, the isolate was recovered again in a rectal swab with the same susceptibility profile.

**Table 1.** Antimicrobial susceptibility and carbapenemase detection results for *Escherichia coli* isolates harbouring *bla*_KPC-2_ and *bla*_KPC-14_ genes

	*Escherichia coli bla*_KPC-2_	*Escherichia coli bla*_KPC-14_
MIC, mg/L		
Piperacillin/tazobactam	>16 (R)	≤8 (S)
Meropenem	>8 (R)	≤0.12 (S)
Imipenem	>4 (R)	≤1 (S)
Ertapenem	>1 (R)	≤0.12 (S)
Ceftazidime/avibactam	≤2 (S)	>8 (R)
Imipenem/relebactam	0.125 (S)	0.19 (S)
Aztreonam/avibactam	0.064 (S)	0.75 (S)
Cefiderocol	0.25 (S)	8 (R)
Disc diffusion, mm		
Cefiderocol	30 (S)	17 (R)
KPC detection		
Immunochromatography	Positive	Negative
Boronic acid synergy	Positive	Negative
MDR Direct Flow Chip Kit	Positive	Positive
Xpert Carba-R	Positive	Positive
GenBank accession no.	SRX32697708	SRX32697709

KPC, *Klebsiella pneumoniae* carbapenemase; R, resistant; S, susceptible.

The patient was readmitted days later for a second infusion following the failure of the first transplant. Subsequent rectal and pharyngeal swabs were negative until 10 days later, when the KPC-producing *E. coli* was re-isolated from surveillance samples, a urine culture and a catheter blood culture, showing the same susceptibility profile as the first one. Treatment with cefiderocol was initiated for 8 days. During this period, no microorganisms were isolated from either epidemiological or clinical samples. Immediately after completing the treatment and before hospital discharge, a final surveillance culture was taken, isolating an *E. coli* that grew only on the ESBL plate, but the MDR kit detected a *bla*_KPC_ gene directly from the rectal/pharyngeal swabs. This isolate was resistant to ceftazidime/avibactam and cefiderocol but susceptible to carbapenems. MDR kit and Xpert^®^ Carba-R (Cepheid^®^) tests from the isolate were positive for KPC but the carbapenemase immunochromatography test was negative, as was the boronic acid synergy test (Table 1). These failures in carbapenemase detection by some phenotypic methods have already been described for certain ceftazidime/avibactam-resistant KPC variants in *K. pneumoniae.*^6,7^ Next-generation sequencing (NGS) was performed on the patient’s first KPC *E. coli*–positive sample and on the ceftazidime/avibactam- and cefiderocol-resistant one. NGS was performed using the Illumina iSeq^™^ 100 system and *de novo* assembly with the SPAdes tool (Galaxy Version 4.2.0 + galaxy0). Identification was performed using pubMLST (https://pubmlst.org/bigsdb?db = pubmlst_rmlst_seqdef_kiosk) and resistance genes were identified with ResFinder version 4.7.2 (http://genepi.food.dtu.dk/resfinder). The first isolate carried the *bla*_KPC-2_ gene, while the second encoded the *bla*_KPC-14_ gene. In our case, this KPC mutation was detected following the use of cefiderocol.

Microbiology laboratories use various phenotypic tools for the rapid detection of carbapenemases, such as immunochromatography. While these methods typically offer reliable detection for common carbapenemases, their efficacy may be compromised when confronting mutated carbapenemase variants. Therefore, it seems indispensable to complement detection with PCR multiplex platforms and ultimately with NGS to determine specific carbapenemase variants. Our case highlights that rapid genotypic detection directly from rectal and pharyngeal swabs was key to detecting this mutated carbapenemase variant. It is also indispensable to include ceftazidime/avibactam in susceptibility panels, as this allows for the detection of these ‘hidden’ carbapenemases. Although the *bla*_KPC-14_ variant is strongly associated with ceftazidime/avibactam resistance, its role in cefiderocol resistance is complex. Comparative genomic analysis via Snippy, Prokka and IGV between the KPC-14 and KPC-2 strains revealed four genetic variations: a six-nucleotide in-frame deletion located precisely within the *bla*_KPC_ gene, and three SNPs. One of these SNPs mapped to the inner membrane protein gene *yeeA*. These findings suggest that the cefiderocol resistance could be driven by the enhanced enzymatic action of the novel KPC-14 variant, likely acting in synergy with chromosomal adaptations altering bacterial permeability via the inner membrane protein mentioned.

Although the mechanistic basis for the selection of *bla*_KPC_ variants has been confirmed *in vitro* in recombinant *E. coli* strains, showing an increase in carbapenem susceptibility,^8^ to the best of our knowledge this is the first clinical report of *in vivo* selection of a KPC-14-producing *E. coli* isolate. The finding is of particular relevance as it broadens the alert regarding these mutations to bacterial species other than *K. pneumoniae*.
